# Health System’s Role in Facilitating Health Service Access among Persons with Spinal Cord Injury across 22 Countries

**DOI:** 10.3390/ijerph20116056

**Published:** 2023-06-05

**Authors:** Olena Bychkovska, Vegard Strøm, Piotr Tederko, Julia Patrick Engkasan, Alvydas Juocevičius, Linamara Rizzo Battistella, Mohit Arora, Christoph Egen, Armin Gemperli

**Affiliations:** 1 Swiss Paraplegic Research, Guido A. Zäch Institute, 6207 Nottwil, Switzerland; 2 Department of Health Sciences and Medicine, University of Lucerne, 6002 Lucerne, Switzerland; 3 Department of Research, Sunnaas Rehabilitation Hospital, 1453 Nesoddtangen, Norway; 4 Department of Rehabilitation, Medical University of Warsaw, 02-091 Warsaw, Poland; 5 Department of Rehabilitation Medicine, Universiti Malaya, Kuala Lumpur 50603, Malaysia; 6 Faculty of Health Sciences, Klaipeda University, 92255 Klaipeda, Lithuania; 7 Faculty of Medicine, University of São Paulo, São Paulo 01246-903, Brazil; 8 John Walsh Centre for Rehabilitation Research, The Kolling Institute, St Leonards 2065, Australia; 9 Faculty of Medicine and Health, University of Sydney, Sydney 2006, Australia; 10 Department of Rehabilitation Medicine, Hannover Medical School, 30625 Hannover, Germany; 11 Center for Primary and Community Care, University of Lucerne, 6002 Lucerne, Switzerland

**Keywords:** health systems, health services, health service access, access barriers, disability, spinal cord injury, country comparison

## Abstract

(1) Background: Despite efforts to improve access to health services, between- and within-country access inequalities remain, especially for individuals with complex disabling conditions like spinal cord injury (SCI). Persons with SCI require regular multidisciplinary follow-up care yet experience more access barriers than the general population. This study examines health system characteristics associated with access among persons with SCI across 22 countries. (2) Methods: Study data are from the International Spinal Cord Injury Survey with 12,588 participants with SCI across 22 countries. Cluster analysis was used to identify service access clusters based on reported access restrictions. The association between service access and health system characteristics (health workforce, infrastructure density, health expenditure) was determined by means of classification and regression trees. (3) Results: Unmet needs were reported by 17% of participants: lowest (10%) in Japan, Spain, and Switzerland (cluster 1) and highest (62%) in Morocco (cluster 8). The country of residence was the most important factor in facilitating access. Those reporting access restrictions were more likely to live in Morocco, to be in the lowest income decile, with multiple comorbidities (Secondary Conditions Scale (SCI-SCS) score > 29) and low functioning status (Spinal Cord Independence Measure score < 53). Those less likely to report access restriction tended to reside in all other countries except Brazil, China, Malaysia, Morocco, Poland, South Africa, and South Korea and have fewer comorbidities (SCI-SCS < 23). (4) Conclusions: The country of residence was the most important factor in facilitating health service access. Following the country of residence, higher income and better health were the most important facilitators of service access. Health service availability and affordability were reported as the most frequent health access barriers.

## 1. Introduction

There is a link between the characteristics of a health system, such as the provision, organization, distribution of resources, and health service access, as an outcome a system is trying to achieve [1,2]. Despite making systemic efforts to improve access, between- [3] and within-country [4,5] inequalities persist. Health systems continue to be inequitable, as vulnerable populations face more barriers and less beneficial health outcomes than the general population [6]. Persons with complex health conditions and, thus, high healthcare needs are less likely to receive comprehensive care than those with better health status [6,7,8,9].

Persons with chronic spinal cord injury (SCI) are an illustrative case to understand the situation with health service access for those with high service needs. SCI is damage to the spinal cord as a result of traumatic (e.g., traffic accidents, falls, violence, occupational or sports injuries) or non-traumatic etiology (e.g., tumors, infectious or musculoskeletal diseases). It is a complex and costly health condition that primarily affects men and the elderly and is often accompanied by secondary health conditions [10,11]. Service access was found to be a strong predictor of quality of life for persons with SCI [12], as this condition requires frequent utilization of multidisciplinary services across various settings [10,13,14]. Persons with chronic SCI experience more difficulties accessing services compared to the general population [15,16,17]. The primary care services for persons with chronic SCI are not as comprehensive as for the general population [18,19], nor are they as accessible as acute and rehabilitation services for those recently injured and having an acute SCI [20].

Health service access manifests itself in distinct dimensions: acceptability, approachability, availability and accommodation, affordability, and appropriateness [21,22]. Many access barriers to healthcare are common between persons with SCI and the general population (e.g., workforce quantity and distribution, healthcare financing, including insurance arrangements, and waiting time [1]), while others are more specific to persons with SCI (e.g., inappropriate medicine or medical equipment [23,24,25], lack of health providers’ experience in SCI management [19,24,25,26,27,28]). Overall, health systems of low- and middle-income countries have more capacity-related barriers, which are further amplified by contextual factors [1], e.g., inaccessible transportation to services [17,29,30]. Along with these systemic factors, persons with certain predisposing [1] socio-demographic and health characteristics (women [15,16], persons living in rural areas [31], with immigrant background [14], lower income or lower health status [9]) are more likely to have restricted access to services [6].

Few studies explored the effect of the health system on access to services for persons with SCI [32], focusing on access to primary care [31] or other healthcare settings in a specific geographical region [1]. This study aims to identify health system characteristics associated with access to health services among persons with SCI across 22 countries. The specific objectives are: (1) to develop a classification of countries with regards to health service access in order to identify common barriers for persons with SCI; (2) to determine the association of health system characteristics with access; (3) to examine how this association is modified by socio-demographic and health status characteristics. The hypothesis is that access to health services among persons with SCI is related to health system characteristics [2,22].

## 2. Materials and Methods

### 2.1. Data Collection and Sampling

Data from the International Spinal Cord Injury (InSCI) (2017–2019) international cross-sectional questionnaire survey [33] was used in this study. InSCI is the primary phase of the International Learning Health System for Spinal Cord Injury Study (LHS-SCI) established with the endorsement of the World Health Organization (WHO), the International Spinal Cord Society (ISCoS) and the International Society for Physical and Rehabilitation Medicine (ISPRM), following the WHO’s Global Disability Plan [34]. InSCI is the first community-based survey to describe the experience of persons with SCI living in the community and the perceived societal response to their needs. The plan is to repeat the survey every five years following the initial 2017–2019 survey in order to provide evidence for strengthening health services for persons with SCI and the general population [35].

Data were collected by a study center of each participating country with general coordination done by the InSCI Study Center at Swiss Paraplegic Research in Nottwil, Switzerland [33]. InSCI sampling frames were formed from the available information sources on persons with SCI in each country: national registries, clinical records of specialized rehabilitation centers, academic or trauma hospitals, and membership registries of organizations for persons with disability or insurance agencies. The questionnaire contained 125 items and had multiple response options: online or paper-pencil questionnaires and face-to-face or telephone interviews.

Inclusion criteria for participation were being an adult 18 years old and older with a traumatic or non-traumatic SCI, who has completed initial acute care or rehabilitation and who resided in one of the following 22 countries: Australia, Brazil, China, France, Germany, Greece, Indonesia, Italy, Japan, Lithuania, Malaysia, Morocco, the Netherlands, Norway, Poland, Romania, South Africa, South Korea, Spain, Switzerland, Thailand, and the United States (USA).

Each participating country attained ethical approval. Each participant or participant’s authorized representative signed an informed consent form to participate in the study. All of the de-identified data were stored in a protected central database [33]. Data for this study were obtained from the InSCI Study Center after submitting a project proposal, which was reviewed and accepted by the InSCI Scientific Committee.

### 2.2. Data Analysis and Management

#### 2.2.1. Health System Characteristics

Data for describing the health system were extracted from data resources by the World Health Organization (WHO) [36] and the Organization for Economic Co-operation and Development (OECD) [37] (Supplementary Table S1). Previous publications extensively described the national and cross-country context along with the health systems and economic characteristics of InSCI countries [12,38].

The *number of SCI specialized centers* was derived from InSCI country reports and InSCI network experts’ opinions. *Health workforce* and *infrastructure density* were measured as the number of personnel or beds per 10,000 residents [36]. *The Healthcare Access and Quality Index* (range: 0 (worst score)–100 (best score), median: 62) [3] was based on data from 195 countries on amenable mortality from 32 causes of death that could have been avoided by accessing medical care. *Universal Health Coverage (UHC) Index of Service Coverage score* (range: 0 (worst score)–100 (best score), global score: 67) [39,40] is an indicator of essential health services coverage based on 14 service coverage indicators. The *percentage of the population availing of social protection* [37] denotes the share of the population covered by governmental or private health insurance. Current *health expenditure* as a share of gross domestic product (GDP) is the share of all-actors expenditure on health, while domestic general government health expenditure as a percentage of GDP is the share of expenditure on health by the government. Out-of-pocket expenditure as a percentage of current health expenditure is the share of expenditure on health paid by households directly out-of-pocket [36].

#### 2.2.2. Health Service Access

Self-reported unmet healthcare needs were defined as not getting the needed health service in the last twelve months. The specific reasons for not getting the health service were categorized across five access dimensions: acceptability (the person was previously badly treated), approachability (the person did not know where to go or considered they were not sick enough to require a service), availability and accommodation (there was no service available; there was no transport available to the service; the person was denied a service; the person could not take time off work or other commitments), affordability (the person could not afford the cost of the service or the transportation to the service), appropriateness (the person considered the health provider’s drugs or equipment were inadequate or provider’s skills were inadequate) [21,22].

#### 2.2.3. Socio-Demographic and Health Status Characteristics

Education was measured in line with the International Standard Classification of Education, summing up the total years of formal education, including school and vocational training [41,42]. Income represented equivalent total household income translated to country-specific income deciles. The deciles divide the population into ten income-ranked groups [41,43].

A summarized score of secondary health conditions severity was based on a modified version of the Spinal Cord Injury Secondary Health Conditions Scale (SCI-SCS) (range: 0–56) [44] on experiencing health problems in the last three months. It included the following 14 health conditions: sleep problems, bowel dysfunction, urinary tract infections, bladder dysfunction, sexual dysfunction, contractures, muscle spasms or spasticity, pressure sores or decubitus, respiratory problems, injury caused by a loss of sensation, circulatory problems, autonomic dysreflexia, postural hypotension, and pain. Each health condition was rated from 0 (no problem) to 4 (extreme problem) for all countries except for Switzerland, where a four-point scale was used. The answers in the four-point scale were weighted as 0, 1.3, 2.3 and 4, respectively, to align with the 0 to 4 weighting in the five-point scale.

The Spinal Cord Independence Measure (SCIM-III self-report) score (0–66) was used as a measure of independence in activities of daily living. It contained the following questionnaire items: eating and drinking, washing the upper body and head, washing the lower body, dressing the upper body, dressing the lower body, grooming, use of an indwelling catheter, intermittent bladder catheterization, use of external drainage instruments, bowel assistance, bowel movement, fecal incontinence, toileting, turning the upper body in bed, turning the lower body in bed, sitting up in bed, doing push-ups in a chair or a wheelchair, transfer from bed to a wheelchair, moving around moderate distances. The recording and creation of a single score by summing up the items was done according to Fekete et al. [45].

### 2.3. Statistical Analysis

By means of a cluster analysis [46], the participating countries were classified regarding access restrictions reported by persons with SCI. Hierarchical cluster analysis was performed based on a dissimilarity matrix on Gower distance and Ward’s linkage [47]. To confirm the results of this analysis, additional cluster analyses were also performed based on other linkage types (single, average, centroid, and complete) as well as 5–10 k k-means clustering, checked by Calinski-Harabasz pseudo-F index and Duda-Hart Je(2)/Je(1) index stopping rules.

To identify the health system and individual characteristics associated with access, classification and regression trees (CART) were developed. For each of the six outcomes (unmet needs, acceptability, approachability, availability and accommodation, affordability, and appropriateness), two models were built: one with and one without countries as predictors. Predictors in the tree were the following: (1) health system characteristics as described above (workforce and infrastructure density, Healthcare Access and Quality Index, UHC Index of Service Coverage score, government health expenditure, out-of-pocket health expenditure, insurance coverage); (2) socio-demographic characteristics (sex, age, migration background, living arrangement, assistance received with day-to-day activities from family, friends or professionals, education, income, having paid work, difficulties using public, private and long-distance transportation); (3) health status and SCI characteristics (level: tetra- or paraplegia, completeness: complete or incomplete, etiology: traumatic or nontraumatic, years lived with injury, SCI-SCS index, SCIM index, confidence in the ability to maintain good health, self-rated health, satisfaction with own health). To better describe the results, we use two groups of respondents: the first group comprises those respondents that were more likely to report access restrictions, and the second group is those who were less likely to report restrictions.

All analyses had a descriptive and exploratory purpose. As the CART algorithm does not allow missing values, missing predictors were imputed using random forest imputation [48]. Data preparation and analysis were conducted using Stata 16.1 (StataCorp LLC, College Station, TX, USA) and R 6.2.0 (R Foundation for Statistical Computing, Vienna, Austria) with packages missForest [48] (data imputation) and rpart [49] (CART).

## 3. Results

### 3.1. Health System Characteristics

The countries with a number of specialized centers for SCI management higher than 10 were France, Germany, Spain, and the USA (Supplementary Table S1). The highest number of hospital beds per 10,000 residents was found in Japan (131), and the lowest was in Indonesia and Morocco (10 each). The following figures illustrate the number of health professionals per 10,000 residents. Countries with the highest number of doctors were Norway (47) and Lithuania (45), and the lowest number in Indonesia (4). The number of nursing and midwifery personnel was the highest in Norway (184) and Switzerland (183) and the lowest in Morocco (14). The number of physiotherapists was highest in Norway (24), while no physiotherapists were recorded in China, Indonesia, Malaysia, and Morocco, possibly due to a difference in professions classification. The number of pharmacists was the highest in Japan (19) and the lowest in Indonesia and The Netherlands (2). Dentists’ number was the highest in Brazil and Greece (13) and the lowest in Indonesia, Morocco, and South Korea (1) [36].

The Healthcare Access and Quality Index [3] score was the highest in Norway (97), closely followed by Switzerland, Australia, and the Netherlands (96 each). The lowest index was in Indonesia (44), South Africa (50), Morocco (58), Brazil (64), and Malaysia (68). Australia and South Korea had the highest (87) Universal Health Coverage (UHC) Index of Service Coverage score [39,40], and Indonesia had the lowest (59). The percentage of the population having social protection was the lowest in the USA (36%) and Morocco (42%) [37]. The percentage of the population with household expenditures on health greater than 10% of total household expenditure was the highest in China (24%), Morocco (21%) and lowest in South Africa (1%), Germany, Malaysia and Thailand (2%). Out-of-pocket expenditure as a percentage of current health expenditure was the highest in Morocco (54%) and the lowest in South Korea (6%). Countries that spent the most on healthcare were France, Germany, Japan, the Netherlands, and the USA, while the lowest expenditure was in Indonesia [36].

### 3.2. Socio-Demographic and Health Status Characteristics of Study Participants

The analysis was conducted among 12,588 participants. The survey response rates were only available for countries with predefined sampling frames: South Africa (response rate of 54%), Norway (42%), Switzerland (39%), the Netherlands (33%), Germany (32%), Poland (32%), Australia (27%), and China (23%) [41]. The sample was predominantly male (73%), with an average age of 51 years. The majority of the participants were living with others (77%) (Supplementary Table S2). The majority of the participants had paraplegia (61%), with an incomplete lesion (60%) and traumatic etiology (80%) for 13 years on average (Supplementary Table S3). The average SCI-SCS score was 17 (lowest in Brazil and China (11), highest in South Korea (27)), while the average SCIM score was 40 (lowest in South Korea (28) and highest in Norway (50)). Socio-demographic characteristics of the study participants by country were described by Fekete et al. [41].

### 3.3. Health Service Access

Across all countries, 17% (95% confidence interval: 16.6–17.9%) of participants indicated that in the twelve months preceding the study, they needed a healthcare service but did not receive it. The largest share of those indicating this unmet need was in Morocco (62%), South Africa (28%), South Korea (27%), Brazil and China (24% each), and the lowest in Switzerland and Spain (7%). The most common reason for not receiving healthcare service was the unavailability of services (9%), followed by unaffordability (7%), inappropriateness and unapproachability (4% each). Issues with acceptability were reported by 2% of the participants. The most frequently reported availability issues were in Morocco (38%), South Africa (20%), Poland (14%), South Korea (12%), Germany and China (10%), and least frequently in Norway and Spain (3%). The most frequently reported affordability restrictions were reported in Morocco (53%), Brazil and China (13% each) and least frequently reported in France, Japan, the Netherlands, and Spain (1% each). In terms of approachability issues, the highest share was reported in China (10%), Morocco (9%), and the lowest in Brazil, Italy, and Thailand (1% each). Appropriateness and acceptability restrictions were most frequently reported in Morocco (8%) and least in France, Japan, and Spain (1% and below).

### 3.4. Characteristics of Health Service Access Clusters

The cluster analysis identified seven health service access clusters (Table 1).

*Cluster 1 (Japan, Spain, and Switzerland).* The cluster with the lowest level of unmet needs (10%) was almost half as low as the overall average (17%). Issues in all access dimensions were less frequent than the average.

*Cluster 2 (Indonesia, Italy, Thailand, and the USA).* This cluster’s frequency of unmet needs (12%) was below the overall average. Problems with appropriateness were more frequent than the overall average (6% versus 4%). Problems with acceptability and affordability were 1% more frequent than the overall average. Problems in all other accessibility dimensions were below all clusters and were less frequent than the overall average.

*Cluster 3 (Australia, France, Norway, and the Netherlands).* In this cluster, the frequency of unmet needs (12%) was below the overall average and similar to clusters 2 and 4. Restrictions across all access dimensions were reported as less frequent than average, except for problems with appropriateness (5% versus an average of 4%).

*Cluster 4 (Germany, Greece, Lithuania, and Romania).* Similar to clusters 2 and 3, the reported frequency of unmet needs was 12%, below the overall average. Restrictions in all access dimensions were less frequent than the overall average.

*Cluster 5 (Brazil and China)*. In this cluster, the reported frequency of unmet needs (24%) was 7% higher than the overall average. The frequencies of reported problems in affordability, approachability and acceptability were higher than the cluster level, while overall average. The frequency of affordability restrictions was similar to the overall.

*Cluster 6 (Malaysia, Poland, South Africa, and South Korea).* The frequency of unmet needs (26%) was 9% higher in this cluster than the overall average. Restrictions in all access dimensions were more frequent than the overall average.

*Cluster 7 (Morocco).* This cluster’s reported frequency of unmet needs (62%) was almost four times the overall average. Restrictions in affordability were reported eight times as frequently as the overall average, while problems with availability and acceptability were reported four times, and those regarding appropriateness and approachability were twice that of the overall average. Compared to the overall average, this cluster had more frequently reported problems across all access dimensions.

### 3.5. Association between Health Service Access, Health System and Individual Characteristics

Residence country was identified as the most important factor associated with reporting access barriers in both classification trees with and without country variables (Table 2). Those that reported unmet needs, availability and affordability restrictions were more likely to live in Morocco, to be in the lowest income decile and to have lower health status and more profound functional limitations (SCI-SCS score higher than 29 and SCIM score lower than 53). Those who had the least problems with health service access tended to be healthier (SCI-SCS score lower than 23) and reside in all other countries except Morocco. In this second group, those residing in Brazil, China, Malaysia, Poland, South Africa, or South Korea were more likely to have unmet needs and those in Indonesia or Thailand to have affordability restrictions.

## 4. Discussion

This study examined the association between health system characteristics and access to health services among persons with SCI and to what extent this association is modified by socio-demographic and health status characteristics. We found that country of residence was the most important factor associated with access to health services. No health system characteristic related to access was identified. Factors, such as the number of doctors or nurses, were important in defining access to health services, yet they act mainly as surrogates for the countries’ overall characteristics. Even though income and health status were statistically significant in predicting unmet needs, personal characteristics played a less important role in comparison to the country factor.

It has been established before that health service access for persons with SCI is highly dependent on larger contextual factors, such as transportation and social attitudes [1,12,17,29,50]. Hence, the larger economic, social and cultural context [2] may be more important for facilitating access to health services than the characteristics of the health system. Such contextual factors are further modified by predisposing [1] characteristics; hence, in this study, individuals with low income [9,16,17,51,52,53] and lower health status [9,15,54,55] reported more access restrictions. Those with low health status are likely also to have low income, which in turn may lead to experiencing more barriers and further health status worsening [56].

The findings of the cluster analysis and the decision trees were consistent in identifying countries with higher reported access restrictions. These countries were Indonesia and Thailand (cluster 2), Brazil, China (cluster 5), Malaysia, Poland, South Africa, South Korea (cluster 6), and Morocco (cluster 7). Countries in these clusters had fewer resources for managing SCI, such as specialized centers. Especially Indonesia and Morocco had fewer hospital beds and health workforce, with particularly visible differences in the medical and therapeutic personnel density, such as physiotherapists or dentists. These countries had lower governmental expenditure on health (overall below 5%, with 1% in Indonesia and 2% in Malaysia and Morocco) along with higher out-of-pocket expenditures (overall above 30%, with up to 54% in Morocco) [36]. The country scores of the Healthcare Access and Quality Index (all-country average: 62) [3] and UHC Index of Service Coverage (all-country average: 67) [39] were predominantly between 50 and 80, with only Indonesia having lower scores than the all-countries averages (44 and 59 respectively). In countries where SCI persons reported fewer access barriers, this score range was between 82 and 96.

This is the first study to identify health system characteristics associated with access to health care from the perspectives of persons with SCI across 22 countries. In this study, some limitations were present. The health system’s characteristics might not have been comprehensively measured, and the indicators may not have fully captured the impact of the health system. It is challenging to distinguish if the most important factor of residence country in this instance represents a separate effect of its national health system, governance and policies, social, economic, and cultural environment, or interaction of these effects. Self-reported cross-sectional data were used, which might be subject to various biases, for example, recall bias and differences in medical and non-medical expectations from the health system. The data collection methods differed among participating countries, which could have led to a difference in data quality. The sampling frames in most countries were bound to a specific region and did not represent the country entirely. In certain countries, the sampling setting was restricted to rehabilitation facilities (Brazil, Germany, the Netherlands, Norway) or general or acute hospitals (China, Spain). In countries using convenience sampling (Brazil, France, Greece, Indonesia, Italy, Japan, Lithuania, Malaysia, Morocco, Romania, South Korea, Spain, Thailand, USA), bias could result from self-selection with a lower chance for participation from those experiencing more access restrictions. The response rate (27–54%) indicates that those with unmet health needs could be unequally represented as they might have been more challenged to reach out and be included in this study.

## 5. Conclusions

Country of residence was the most important factor in facilitating health service access. Following the country of residence, higher income and better health were the most important facilitators of service access. Health service availability and affordability were reported as the most frequent health access barriers.

## Figures and Tables

**Table 1:** Characteristics of health service access clusters.

	Country	Unmet Needs ^a^ (%)	Availability (%)	Affordability (%)	Approachability (%)	Appropriateness (%)	Acceptability (%)
Total all clusters ^b^		17.4	8.8	7.1	3.8	3.6	2.3
Cluster 1 ^c^	Total	9.5	4.1	1.5	3.4	2.0	0.7
	CH	6.9	2.6	2.1	1.7	1.6	0.7
	ES	6.7	3.4	1.0	2.9	0.5	0.2
	JP	12.6	6.3	0.7	6.3	1.3	0.7
Cluster 2 ^c^	Total	11.5	11.2	8.3	2.4	5.8	2.7
	ID	12.4	4.5	5.5	1.5	3.5	0.5
	IT	11.7	5.8	4.9	0.5	1.9	2.9
	TH	10.0	5.6	3.1	1.3	2.2	0.9
	USA	11.8	6.4	3.0	1.5	3.9	1.0
Cluster 3 ^c^	Total	11.8	4.0	2.3	2.8	4.8	1.4
	AU	16.1	6.4	5.1	4.4	4.5	2.2
	FR	10.4	1.7	1.2	2.2	5.3	0.2
	NL	11.5	5.0	1.2	2.7	5.4	1.5
	NO	9.2	3.0	1.6	1.8	3.9	1.5
Cluster 4 ^c^	Total	12.4	7.4	3.7	3.2	3.6	1.8
	DE	12.9	9.7	2.6	3.2	2.5	1.1
	GR	11.0	6.5	4.0	3.5	4.5	0.5
	LT	13.3	6.0	5.5	2.8	4.6	1.8
	RO	12.5	7.4	2.8	3.2	2.8	3.7
Cluster 5 ^a^	Total	24.3	8.9	13.1	5.2	3.1	6.2
	BR	24.4	8.0	13.4	0.5	2.5	4.0
	CN	24.1	9.7	12.8	9.8	3.7	8.3
Cluster 6 ^c^	Total	25.5	14.6	8.1	6.0	4.1	2.8
	MY	22.2	12.1	7.7	6.4	2.7	1.0
	PL	25.3	14.2	8.4	8.9	6.4	5.8
	ZA	27.5	20.0	8.5	1.5	2.0	1.5
	KR	27.0	12.0	7.6	7.2	5.4	2.8
Cluster 7 ^c^	MA	62.3	37.7	52.7	9.1	7.5	7.5

^a^ Unmet need: needing but not receiving a healthcare service. The margin of error for the reported health service unmet needs is below 0.5% with a 95% confidence level. ^b^ Missing values are counted as reporting no access restriction ^c^ Cluster 1: CH—Switzerland, ES—Spain, JP—Japan; Cluster 2: ID—Indonesia, IT—Italy, TH—Thailand, USA—the United States; Cluster 3: AU—Australia, FR—France, NL—the Netherlands, NO—Norway; Cluster 4: DE—Germany, GR—Greece, LT—Lithuania, RO—Romania; Cluster 5: BR—Brazil, CN—China; Cluster 6: MY—Malaysia, PL—Poland, ZA—South Africa, KR—South Korea; Cluster 7: MA—Morocco.

**Table 2:** Association between health service access, health system and individual characteristics.

Subgroup 1: Persons Likely to Report Service Access Barriers	N ^a^	n (%) ^b,c^	Subgroup 2: Persons Likely to Not Report Service Access Barriers	N ^a^	n (%) ^b,c^
––––––––––––––––––––––––Unmet needs (with country)––––––––––––––––––––––––					
All participants	12,588	2169 (17.2%)	All participants	12,588	10,419 (82.8%)
→ Country: BR, CN, MY, MA, PL, ZA, KR ^d^	4298	1222 (28.4%) *	→ All other countries	8290	7343 (88.6%) *
→ Country: MA	385	240 (62.3%) *	→ SCI-SCS ^e^ < 24	6267	5778 (92.2%) **
→ Income decile 1	261	182 (69.7%)			
––––––––––––––––––––––Unmet needs (without country)–––––––––––––––––––––––					
All participants	12,588	2169 (17.2%)	All participants	12,588	10,419 (82.8%)
→ Number of nurses < 22	385	240 (62.3%) *	→ Number of nurses ≥ 22	12,203	10,274 (84.2%) *
→ Income decile 1	261	182 (69.7%)	→ SCI-SCS < 23	9026	7971 (88.3%) **
→ SCI-SCS ≥ 3.5	251	180 (71.7%)			
–––––––––––––––––––––––––Availability (with country)–––––––––––––––––––––––––					
All participants	12,588	1075 (8.5%)	All participants	12,588	11,513 (91.5%)
→ Country: MA	385	240 (62.3%) *	→ Country: all except MA	12,203	11,273 (92.4%) *
→ SCI-SCS ≥ 29	38	28 (73.7%) ***	→ SCI-SCS < 27	9943	9392 (94.5%) **
–––––––––––––––––––––––Availability (without country)–––––––––––––––––––––––					
All participants	12,588	1075 (8.5%)	All participants	12,588	11,513 (91.5%)
→ Number of nurses < 22	385	240 (62.3%) *	→ Number of nurses ≥ 22	12,203	11,273 (92.4%) *
→ SCI-SCS ≥ 29	38	28 (73.7%) ***	→ SCI-SCS < 27	9943	9392 (94.5%) **
–––––––––––––––––––––––Affordability (with country)–––––––––––––––––––––––––					
All participants	12,588	835 (6.6%)	All participants	12,588	11,753 (93.4%)
→ Country: MA	385	203 (52.7%) *	→ Country: all except MA	12,203	11,571 (94.8%) *
→ Income decile 1	261	158 (60.5%) *	→ Country: all except BR, CN, MY, PL, ZA, KR ^d^	8290	8048 (97.1%) *
→ SCIM ^f^ < 53	229	149 (65.1%) *	→ SCI-SCS < 23	6257	6155 (98.4%)
–––––––––––––––––––––––Affordability (without country)––––––––––––––––––––––					
All participants	12,588	835 (6.6%)	All participants	12,588	11,753 (93.4%)
→ Number of nurses < 22	385	203 (52.7%) *	→ Number of nurses ≥ 22	12,203	11,571 (94.8%) *
→ Income decile 1	261	158 (60.5%) *	→ Number of doctors ≥ 24	7769	7548 (97.2%) **
→ SCIM < 53	229	149 (65.1%) *			

Subgroups were identified using classification and regression tree analysis. Subgroups are shown cumulatively, such that each subgroup is nested within the subgroup from the row above. * Significant predictors after cross-validation. ** Terminal node. *** is the combination of * and **. ^a^ N: number of participants in the respective subgroup. ^b^ n: number of participants who reported unmet needs or access restrictions in the respective node. ^c^ %: percentage of participants who reported unmet needs or access restrictions in the respective node. ^d^ BR—Brazil, CN—China, MY—Malaysia, MA—Morocco, PL—Poland, ZA—South Africa, KR—South Korea. ^e^ Spinal Cord Injury Secondary Health Conditions Scale (range: 0–56) based on a self-rated question about 14 health problems. ^f^ Spinal Cord Independence Measure (range: 0–66): a measure of independence in activities using daily living scores.

## Data Availability

The data supporting this study’s findings are available from the InSCI Study Group, but restrictions apply to the availability of these data, which were used under license for the current study and are not publicly available. Data are, however, available from the authors upon reasonable request and with permission of the InSCI Study Group.

## Supplementary Materials

The following supporting information can be downloaded at: https://www.mdpi.com/article/10.3390/ijerph20116056/s1, Table S1: Health system characteristics of InSCI countries; Table S2: Socio-demographic characteristics of study participants; Table S3: Health status characteristics of study participants. References [57,58,59,60,61,62,63,64,65] are in Supplementary Materials.
