# Supplementary material for: Health System’s Role in Facilitating Health Service Access among Persons with Spinal Cord Injury across 22 Countries
Source: Int J Environ Res Public Health. 2023 Jun 5;20(11):6056. doi: 10.3390/ijerph20116056 (PMC10252714; doi:10.3390/ijerph20116056)
Supplement: Supplementary file 1 [file ijerph-20-06056-s001.zip › Supplementary Table S1.pdf]

Supplementary Table S1. Health system characteristics of InSCI countries

Country[a]	AU	BR	CN	FR	DE	GR	ID	IT	JP	LT	MY	MA	NL	NO	PL	RO	ZA	KR	ES	CH	TH	USA
SCI specialized centers[b]	≤ 10	≤ 10	0	> 10	> 10	0	0	0	≤ 5	0	0	0	0	≤ 5	≤ 5	0	0	0	> 10	≤ 5	0	> 10
Hospital beds[c]	38	21	43	60	80	42	10	32	131	64	19	10	33	36	66	69	123	23	30	47	21	29
Medical doctors[c]	37	22	20	33	43	62	4	40	25	45	19	7	36	47	24	30	24	8	39	43	8	26
Nursing and midwifery personnel[c]	131	74	31	118	142	37	40	63	120	101	35	14	116	184	69	74	82	50	61	183	32	157
Physiotherapists[c]	9	8	0	13	23	8	0	10	5	12	0	0	19	24	7	1	7	1	11	13	1	7
Pharmacists[c]	9	7	3	11	7	11	2	12	19	10	4	3	2	8	8	9	7	3	12	7	4	9
Dentists[c]	6	13	5	6	9	13	1	8	8	10	3	1	6	9	4	8	5	1	8	5	2	6
Healthcare Access and Quality Index[d]	96	64	78	92	92	90	44	95	94	80	68	58	96	97	82	78	50	90	92	96	69	89
Percentage of population that has social protection[e]	100	100	97	100	89	100	59	100	100	94	100	42	100	100	93	100	94	100	99	100	98	36
UHC Index of Service Coverage[f]	87	75	82	84	86	78	59	83	85	70	76	73	86	86	74	71	87	67	86	87	83	83
Population with household health expenditures > 10% of total household expenditure or income[g]	3	12	24	1	2	17	5	9	10	13	2	21	5	5	13	13	12	1	7	20	2	4
Out-of-pocket expenditure as percentage of current health expenditure (%)[h]	18	25	36	10	13	35	37	24	13	33	34	54	11	15	23	21	33	6	22	26	12	11
Health expenditure as percentage of gross domestic product (%)[i]	9	10	5	11	11	8	3	9	11	7	4	5	10	10	7	5	7	9	9	12	4	17
Domestic general government health expenditure as percentage of gross domestic product (%)[j]	6	4	3	9	9	4	1	6	9	4	2	2	7	9	5	4	4	5	6	4	3	9

[a] AU – Australia, BR – Brazil, CN – China, FR – France, DE – Germany, GR – Greece, ID – Indonesia, IT – Italy, JP – Japan, LT – Lithuania, MY – Malaysia, MA – Morocco, NL – the Netherlands, NO – Norway, PL – Poland, RO – Romania, ZA – South Africa, KR – South Korea, ES – Spain, CH – Switzerland, TH – Thailand, USA – the United States

[b] Number of specialized SCI centers according to InSCI Country profiles [57] and experts within the InSCI network

[c] Number per 10,000 population. Data from WHO The Global Health Observatory [58]

[d] Healthcare Access and Quality Index, 2016: personal health-care access and quality index based on mortality from causes amenable to personal health care in 195 countries and territories (0-100). Different health service areas were covered by 32 amenable causes: infectious diseases, maternal and child health; non-communicable diseases; vaccine-preventable diseases; gastrointestinal conditions from which surgery can easily avert death. [59]

[e] Data from OECD data, Percentage of population that has social protection [60], supplemented by indicators from Our World in Data [61]

[f] UHC Index of Service Coverage: "coverage of essential health services (defined as the average coverage of essential services based on tracer interventions that include reproductive, maternal, newborn and child health, infectious diseases, non-communicable diseases and service capacity and access, among the general and the most disadvantaged population). The indicator is an index reported on a unitless scale of 0 to 100, which is computed as the geometric mean of 14 tracer indicators of health service coverage. The tracer indicators are as follows, organized by four components of service coverage: 1. Reproductive, maternal, newborn and child health 2. Infectious diseases 3. Noncommunicable diseases 4. Service capacity and access ". Data from WHO The Global Health Observatory, UHC Index of Service Coverage. [40]

[g] Data from WHO The Global Health Observatory, Population with household health expenditures greater than 10% of total household expenditure or income [63].

[h] Data from WHO The Global Health Observatory, Out-of-pocket expenditure as percentage of current health expenditure (%) [64].

[i] Data from WHO The Global Health Observatory, Current health expenditure as percentage of gross domestic product (%) [65].

[j] Data from WHO The Global Health Observatory, Domestic general government health expenditure as percentage of gross domestic product (%) [66].
