# Supplementary material for: Health System’s Role in Facilitating Health Service Access among Persons with Spinal Cord Injury across 22 Countries
Source: Int J Environ Res Public Health. 2023 Jun 5;20(11):6056. doi: 10.3390/ijerph20116056 (PMC10252714; doi:10.3390/ijerph20116056)
Supplement: Supplementary file 1 [file ijerph-20-06056-s001.zip › Supplementary Table S2.pdf]

Supplementary Table S2. Socio-demographic characteristics of study participants

Country[a]	Total	AU	BR	CN	FR	DE	GR	ID	IT	JP	LT	MY	MA	NL	NO	PL	RO	ZA	KR	ES	CH	TH	USA
Total population, n	12588	1579	201	1354	412	1617	200	201	206	302	218	297	385	260	609	971	216	200	890	417	1530	320	203
Characteristics, n (%)[b]																							
Female	3391 (26.9)	422 (26.7)	42 (20.9)	391 (28.9)	112 (27.2)	447 (27.6)	54 (27.0)	66 (32.8)	53 (25.7)	47 (15.6)	81 (37.2)	61 (20.5)	106 (27.5)	87 (33.5)	192 (31.5)	164 (16.9)	60 (27.8)	50 (25.0)	214 (24.0)	125 (30.0)	440 (28.8)	92 (28.8)	85 (41.9)
Age – mean (min, max)	51.3 (18.0, 96.0)	57.5 (19.0, 94.0)	44.2 (19.0, 86.0)	49.7 (18.0, 91.0)	51.6 (21.0, 93.0)	55.4 (18.0, 91.0)	46.7 (22.0, 82.0)	43.8 (19.0, 78.0)	50.9 (20.0, 83.0)	54.8 (19.0, 85.0)	42.3 (18.0, 70.0)	40.1 (18.0, 75.0)	38.6 (19.0, 89.0)	58.6 (19.0, 90.0)	57.2 (18.0, 96.0)	46.8 (19.0, 87.0)	38.2 (19.0, 74.0)	38.4 (18.0, 72.0)	48.0 (19.0, 82.0)	51.3 (20.0, 89.0)	57.2 (20.0, 93.0)	45.0 (19.0, 79.0)	45.0 (19.0, 76.0)
Migrant background	951 (7.6)	359 (22.7)	0 (0.0)	0 (0.0)	26 (6.3)	111 (6.9)	25 (12.6)	0 (0.0)	14 (6.8)	1 (0.3)	2 (0.9)	3 (1.0)	2 (0.5)	20 (7.7)	46 (7.6)	7 (0.7)	1 (0.5)	7 (3.5)	2 (0.2)	27 (6.5)	281 (18.4)	1 (0.3)	16 (7.9)
Living in institution	413 (3.3)	55 (3.5)	0 (0.0)	32 (2.4)	6 (1.5)	54 (3.3)	1 (0.5)	32 (15.9)	4 (1.9)	5 (1.7)	0 (0.0)	14 (4.7)	3 (0.8)	5 (1.9)	15 (2.5)	38 (3.9)	1 (0.5)	55 (27.5)	9 (1.0)	8 (1.9)	52 (3.4)	23 (7.2)	1 (0.5)
No assistance	3095 (24.6)	428 (27.1)	41 (20.4)	418 (30.9)	120 (29.1)	197 (12.2)	34 (17.0)	60 (29.9)	71 (34.5)	27 (8.9)	43 (19.7)	65 (21.9)	45 (11.7)	112 (43.1)	313 (51.4)	148 (15.2)	31 (14.4)	54 (27.0)	136 (15.3)	109 (26.1)	464 (30.3)	91 (28.4)	88 (43.3)
No education	152 (1.2)	2 (0.1)	4 (2.0)	41 (3.0)	9 (2.2)	0 (0.0)	1 (0.5)	4 (2.0)	0 (0.0)	0 (0.0)	0 (0.0)	0 (0.0)	69 (17.9)	6 (2.3)	0 (0.0)	1 (0.1)	0 (0.0)	0 (0.0)	4 (0.4)	0 (0.0)	0 (0.0)	11 (3.4)	0 (0.0)
Income decile																							
1-3	5578 (44.3)	224 (14.2)	53 (26.4)	379 (28.0)	268 (65.0)	985 (60.9)	118 (59.0)	0 (0.0)	115 (55.8)	162 (53.6)	122 (56.0)	0 (0.0)	339 (88.1)	127 (48.8)	432 (70.9)	580 (59.7)	68 (31.5)	60 (30.0)	577 (64.8)	208 (49.9)	514 (33.6)	216 (67.5)	31 (15.3)
4-7	3380 (26.9)	496 (31.4)	58 (28.9)	659 (48.7)	96 (23.3)	354 (21.9)	53 (26.5)	0 (0.0)	66 (32.0)	5 (1.7)	91 (41.7)	0 (0.0)	40 (10.4)	73 (28.1)	101 (16.6)	213 (21.9)	71 (32.9)	94 (47.0)	171 (19.2)	129 (30.9)	475 (31.0)	76 (23.8)	59 (29.1)
8-10	2085 (16.6)	656 (41.5)	88 (43.8)	316 (23.3)	18 (4.4)	79 (4.9)	26 (13.0)	0 (0.0)	17 (8.3)	112 (37.1)	3 (1.4)	0 (0.0)	6 (1.6)	31 (11.9)	46 (7.6)	127 (13.1)	74 (34.3)	43 (21.5)	103 (11.6)	52 (12.5)	174 (11.4)	19 (5.9)	95 (46.8)
Have no paid work	8046 (63.9)	1085 (68.7)	177 (88.1)	1096 (80.9)	282 (68.4)	1067 (66.0)	164 (82.0)	111 (55.2)	144 (69.9)	186 (61.6)	131 (60.1)	199 (67.0)	357 (92.7)	167 (64.2)	383 (62.9)	607 (62.5)	170 (78.7)	151 (75.5)	628 (70.6)	340 (81.5)	264 (17.3)	222 (69.4)	115 (56.7)
Public transport use																							
No / little problem	4955 (39.4)	875 (55.4)	77 (38.3)	549 (40.5)	205 (49.8)	474 (29.3)	51 (25.5)	59 (29.4)	75 (36.4)	128 (42.4)	75 (34.4)	106 (35.7)	55 (14.3)	129 (49.6)	329 (54.0)	368 (37.9)	35 (16.2)	75 (37.5)	123 (13.8)	228 (54.7)	647 (42.3)	170 (53.1)	122 (60.1)
Severe/ extreme problem	6610 (52.5)	544 (34.5)	123 (61.2)	805 (59.5)	175 (42.5)	969 (59.9)	140 (70.0)	138 (68.7)	121 (58.7)	138 (45.7)	141 (64.7)	175 (58.9)	330 (85.7)	113 (43.5)	261 (42.9)	532 (54.8)	176 (81.5)	125 (62.5)	759 (85.3)	162 (38.8)	484 (31.6)	143 (44.7)	56 (27.6)
Private transport use																							
No / little problem	7860 (62.4)	1192 (75.5)	135 (67.2)	736 (54.4)	274 (66.5)	971 (60.0)	107 (53.5)	118 (58.7)	115 (55.8)	155 (51.3)	162 (74.3)	200 (67.3)	112 (29.1)	207 (79.6)	513 (84.2)	639 (65.8)	112 (51.9)	120 (60.0)	334 (37.5)	307 (73.6)	1015 (66.3)	169 (52.8)	167 (82.3)
Severe/ extreme problem	4043 (32.1)	329 (20.8)	66 (32.8)	618 (45.6)	107 (26.0)	547 (33.8)	84 (42.0)	78 (38.8)	80 (38.8)	111 (36.8)	53 (24.3)	82 (27.6)	273 (70.9)	41 (15.8)	84 (13.8)	299 (30.8)	98 (45.4)	80 (40.0)	543 (61.0)	92 (22.1)	203 (13.3)	143 (44.7)	32 (15.8)
Long-distance transport use																							
No / little problem	6443 (51.2)	1011 (64.0)	84 (41.8)	702 (51.8)	206 (50.0)	894 (55.3)	70 (35.0)	83 (41.3)	67 (32.5)	196 (64.9)	80 (36.7)	117 (39.4)	56 (14.5)	192 (73.8)	419 (68.8)	450 (46.3)	70 (32.4)	86 (43.0)	229 (25.7)	182 (43.6)	947 (61.9)	166 (51.9)	136 (67.0)
Severe/ extreme problem	5710 (45.4)	508 (32.2)	116 (57.7)	652 (48.2)	182 (44.2)	603 (37.3)	119 (59.5)	113 (56.2)	125 (60.7)	93 (30.8)	136 (62.4)	166 (55.9)	329 (85.5)	60 (23.1)	169 (27.8)	488 (50.3)	141 (65.3)	113 (56.5)	643 (72.2)	223 (53.5)	520 (34.0)	152 (47.5)	59 (29.1)

[a] AU – Australia, BR – Brazil, CN – China, FR – France, DE – Germany, GR – Greece, ID – Indonesia, IT – Italy, JP – Japan, LT – Lithuania, MY – Malaysia, MA – Morocco, NL – Netherlands, NO – Norway, PL – Poland, RO – Romania, ZA – South Africa, KR – South Korea, ES – Spain, CH – Switzerland, TH – Thailand, USA – the United States

[b] Missing values: sex: 0.3%, age: 0.6%, migrant background: 1.1%, living arrangement: 1.6%, assistance: 0.9%, education: 1.3%, income: 12.3%, paid work: 7.5%, public transport use: 8.1%, private transport use: 5.4%, long-distance transport use: 3.5%
