# Supplementary material for: Health System’s Role in Facilitating Health Service Access among Persons with Spinal Cord Injury across 22 Countries
Source: Int J Environ Res Public Health. 2023 Jun 5;20(11):6056. doi: 10.3390/ijerph20116056 (PMC10252714; doi:10.3390/ijerph20116056)
Supplement: Supplementary file 1 [file ijerph-20-06056-s001.zip › Supplementary Table S3.pdf]

Supplementary Table S3. Health status characteristics of study participants

Country[a]	Total	AU	BR	CN	FR	DE	GR	ID	IT	JP	LT	MY	MA	NL	NO	PL	RO	ZA	KR	ES	CH	TH	USA
Total population, n	12588	1579	201	1354	412	1617	200	201	206	302	218	297	385	260	609	971	216	200	890	417	1530	320	203
Characteristics, n (%)[b]																							
Tetraplegia	4582 (36.4)	580 (38.9)	81 (40.3)	448 (33.1)	136 (32.9)	758 (46.9)	64 (32.2)	20 (10.3)	53 (26.2)	148 (49.0)	65 (29.8)	88 (29.6)	100 (26.0)	98 (37.7)	239 (39.2)	444 (45.7)	66 (30.6)	77 (38.5)	349 (39.2)	150 (36.0)	458 (29.9)	82 (25.6)	78 (38.4)
Incomplete lesion	7599 (60.4)	1042 (66.0)	159 (79.1)	1009 (74.5)	233 (56.6)	1051 (65.0)	107 (53.5)	116 (57.7)	125 (60.7)	108 (35.8)	55 (25.3)	176 (59.3)	213 (55.3)	183 (70.4)	481 (79.0)	525 (54.1)	145 (67.1)	94 (47.0)	372 (41.8)	226 (54.2)	859 (56.1)	175 (54.7)	145 (71.4)
Nontraumatic etiology	2397 (19.0)	258 (16.3)	60 (29.9)	438 (32.3)	77 (18.7)	327 (20.2)	28 (14.0)	24 (11.9)	61 (29.6)	30 (9.9)	14 (6.4)	43 (14.5)	87 (22.6)	95 (36.5)	183 (30.0)	104 (10.7)	35 (16.2)	15 (7.5)	69 (7.8)	93 (22.3)	312 (20.4)	44 (13.8)	0 (0.0)
Years since injury – mean (min, max)	13.1 (0.0, 81.0)	17.2 (1.0, 73.0)	3.3 (0.0, 33.0)	4.6 (0.0, 54.0)	18.1 (0.0, 69.0)	12.9 (0.0, 61.0)	15.0 (1.0, 45.0)	11.0 (0.0, 42.0)	12.1 (1.0, 47.0)	20.0 (0.0, 58.0)	15.7 (1.0, 47.0)	9.2 (0.0, 47.0)	7.0 (0.0, 48.0)	15.2 (1.0, 61.0)	8.3 (1.0, 45.0)	13.8 (1.0, 58.0)	8.2 (0.0, 39.0)	10.3 (0.0, 49.0)	15.6 (0.0, 63.0)	16.3 (1.0, 65.0)	19.1 (1.0, 81.0)	8.3 (0.0, 48.0)	12.7 (0.0, 60.0)
SCI-SCS[c] – mean (min, max)	17.1 (0.0, 56.0)	18.4 (0.0; 54.0)	11.0 (0.0, 41.0)	11.1 (0.0, 56.0)	14.4 (0.0, 33.0)	19.6 (0.0, 56.0)	14.5 (0.0, 41.0)	13.5 (0.0, 46.0)	18.1 (0.0, 47.0)	17.7 (0.0, 46.0)	18.4 (2.0, 56.0)	15.9 (0.0, 48.0)	17.4 (0.0, 43.0)	14.2 (0.0, 42.0)	15.0 (0.0, 48.0)	19.4 (0.0, 54.0)	16.0 (1.0, 44.0)	13.8 (0.0, 39.0)	26.9 (0.0, 56.0)	16.9 (0.0, 44.0)	18.0 (0.0, 53.0)	12.6 (0.0; 53.0)	15.0 (0.0, 41.0)
SCIM[d] – mean (min, max)	40.0 (0.0, 66.0)	40.6 (1.0, 66.0)	34.0 (0.0, 66.0)	43.9 (0.0, 66.0)	44.8 (5.0, 66.0)	38.1 (0.0, 66.0)	39.1 (1.0, 65.0)	42.2 (0.0, 66.0)	39.8 (0.0, 61.0)	33.7 (1.0, 64.0)	38.2 (1.0, 66.0)	38.0 (1.0, 66.0)	33.8 (0.0, 66.0)	44.7 (1.0, 66.0)	50.4 (3.0, 66.0)	37.9 (0.0, 66.0)	39.5 (5.0, 64.0)	35.7 (1.0, 66.0)	27.6 (0.0, 66.0)	39.7 (1.0, 66.0)	45.9 (1.0, 66.0)	38.5 (0.0, 66.0)	43.4 (7.0, 66.0)
Confidence in maintaining good health																							
No / little	2652 (21.1)	286 (18.1)	42 (20.9)	289 (21.3)	71 (17.2)	480 (29.7)	36 (18.0)	37 (18.4)	56 (27.2)	115 (38.1)	38 (17.4)	34 (11.4)	91 (23.6)	56 (21.5)	128 (21.0)	120 (12.4)	33 (15.3)	21 (10.5)	346 (38.9)	85 (20.4)	234 (15.3)	32 (10.0)	22 (10.8)
Moderate	3954 (31.4)	482 (30.5)	43 (21.4)	485 (35.8)	117 (28.4)	536 (33.1)	66 (33.0)	52 (25.9)	73 (35.4)	114 (37.7)	84 (38.5)	59 (19.9)	118 (30.6)	103 (39.6)	206 (33.8)	303 (31.2)	70 (32.4)	44 (22.0)	334 (37.5)	134 (32.1)	400 (26.1)	74 (23.1)	57 (28.1)
Completely / a lot	5370 (42.7)	742 (47.0)	115 (57.2)	580 (42.8)	203 (49.3)	503 (31.1)	86 (43.0)	105 (52.2)	64 (31.1)	64 (21.2)	94 (43.1)	191 (64.3)	176 (45.7)	91 (35.0)	263 (43.2)	521 (53.7)	109 (50.5)	133 (66.5)	193 (21.7)	184 (44.1)	623 (40.7)	210 (65.6)	120 (59.1)
Satisfaction with own health																							
(Very) disappointed	3658 (29.1)	417 (26.4)	45 (22.4)	447 (33.0)	131 (31.8)	570 (35.3)	38 (19.0)	39 (19.4)	53 (25.7)	114 (37.7)	48 (22.0)	40 (13.5)	95 (24.7)	61 (23.5)	159 (26.1)	398 (41.0)	42 (19.4)	26 (13.0)	461 (51.8)	141 (33.8)	248 (16.2)	44 (13.8)	41 (20.2)
Neither	3557 (28.3)	386 (24.4)	51 (25.4)	525 (38.8)	127 (30.8)	371 (22.9)	51 (25.5)	71 (35.3)	70 (34.0)	107 (35.4)	96 (44.0)	71 (23.9)	111 (28.8)	76 (29.2)	206 (33.8)	292 (30.1)	64 (29.6)	36 (18.0)	251 (28.2)	115 (27.6)	301 (19.7)	127 (39.7)	52 (25.6)
(Very) satisfied	4850 (38.5)	716 (45.3)	105 (52.2)	382 (28.2)	138 (33.5)	633 (39.1)	98 (49.0)	88 (43.8)	71 (34.5)	78 (25.8)	72 (33.0)	177 (59.6)	179 (46.5)	113 (43.5)	237 (38.9)	253 (26.1)	108 (50.0)	137 (68.5)	143 (16.1)	154 (36.9)	715 (46.7)	147 (45.9)	106 (52.2)
Rating of own health																							
Fair / poor	5590 (44.4)	519 (32.9)	64 (31.8)	807 (59.6)	167 (40.5)	679 (42.0)	70 (35.0)	53 (26.4)	88 (42.7)	185 (61.3)	76 (34.9)	89 (30.0)	297 (77.1)	86 (33.1)	228 (37.4)	512 (52.7)	71 (32.9)	50 (25.0)	567 (63.7)	210 (50.4)	613 (40.1)	121 (37.8)	38 (18.7)
Moderate	4853 (38.6)	584 (37.0)	94 (46.8)	442 (32.6)	187 (45.4)	712 (44.0)	60 (30.0)	124 (61.7)	81 (39.3)	76 (25.2)	90 (41.3)	131 (44.1)	65 (16.9)	132 (50.8)	250 (41.1)	369 (38.0)	113 (52.3)	73 (36.5)	238 (26.7)	147 (35.3)	671 (43.9)	140 (43.8)	74 (36.5)
Excellent / very good	1872 (14.9)	410 (26.0)	43 (21.4)	105 (7.8)	47 (11.4)	180 (11.1)	57 (28.5)	18 (9.0)	26 (12.6)	38 (12.6)	49 (22.5)	68 (22.9)	23 (6.0)	34 (13.1)	123 (20.2)	64 (6.6)	30 (13.9)	75 (37.5)	48 (5.4)	54 (12.9)	234 (15.3)	58 (18.1)	88 (43.3)

[a] AU – Australia, BR – Brazil, CN – China, FR – France, DE – Germany, GR – Greece, ID – Indonesia, IT – Italy, JP – Japan, LT – Lithuania, MY – Malaysia, MA – Morocco, NL – Netherlands, NO – Norway, PL – Poland, RO – Romania, ZA – South Africa, KR – South Korea, ES – Spain, CH – Switzerland, TH – Thailand, USA – the United States

[b] Missing values: SCI degree: 2.9%, SCI type: 1.3%, time since sci: 2.7%, SCI-SCS: 14.2%, SCIM: 21.4%, confidence in maintaining good health: 4.9%, satisfaction with own health: 4.2%, rating of own health: 2.2%

[c] Spinal Cord Injury Secondary Health Conditions Scale (range: 0–56) based on self-rated question about 14 health problems

[d] Spinal Cord Independence Measure (range: 0–66): a measure of independence in activities of daily living score
